# The impact of food insecurity on receipt of care, retention in care, and viral suppression among people living with HIV/AIDS in the United States: a causal mediation analysis

**DOI:** 10.3389/fpubh.2023.1133328

**Published:** 2023-08-02

**Authors:** Jacob Bleasdale, Yu Liu, Lucia A. Leone, Gene D. Morse, Sarahmona M. Przybyla

**Affiliations:** ^1^Department of Community Health and Health Behavior, School of Public Health and Health Professions, University at Buffalo, Buffalo, NY, United States; ^2^Department of Public Health Sciences, School of Medicine and Dentistry, University of Rochester, Rochester, NY, United States; ^3^Center for Integrated Global Biomedical Sciences, Department of Pharmacy Practice, School of Pharmacy and Pharmaceutical Sciences, University at Buffalo, Buffalo, NY, United States

**Keywords:** causal mediation analysis, food insecurity, HIV/AIDS, HIV care continuum, people living with HIV/AIDS, United States

## Abstract

**Introduction:**

Attaining The Joint United Nations Programme on HIV/AIDS 95-95-95 targets to end the HIV epidemic by 2030 will require a greater understanding of the underlying mechanisms influencing care engagement among people living with HIV/AIDS (PLWHA). One such mechanism is food insecurity, defined as limited or uncertain access to food. Food insecurity has been shown to significantly impact HIV outcomes. Yet, few studies have examined the mechanisms through which food insecurity may influence these outcomes. We aimed to examine the effects of nutritional, behavioral, and mental health mechanisms through which food insecurity may impact HIV care continuum outcomes: receipt of care, retention in care, and viral suppression.

**Methods:**

We conducted a cross-sectional study of 200 PLWHA in New York State, United States from May-August 2022. Participants were recruited using convenience sampling methods. Multivariable logistic regression models were conducted to examine the associations between food insecurity and care continuum outcomes (receipt of care, retention in care, viral suppression), adjusting for age, race, ethnicity, education, income, and marital status. Individual causal mediation analyses were conducted to assess whether behavioral, mental health, and nutritional mechanisms mediated the hypothesized associations.

**Results:**

The median age of participants was 30 years (IQR: 27-37 years). The majority self-identified as Black (54.0%), male (55.5%) and straight/heterosexual (63.0%). Increasing severity of food insecurity was associated with greater odds of non-retention in care (aOR: 1.35, 95% CI: 1.07, 1.70) and viral non-suppression (aOR: 1.29, 95% CI: 1.08, 1.54). For the impact of food insecurity on non-retention in care, there was an indirect relationship (natural indirect effect; NIE) mediated through Body Mass Index (BMI) (OR_NIE_: 1.08, 95% CI: 1.00, 1.18). For viral non-suppression, there was an indirect relationship mediated through BMI (OR_NIE_: 1.07, 95% CI: 1.00,1.16) and an indirect relationship mediated through depression (OR_NIE_: 1.27, 95% CI: 1.07, 1.47).

**Discussion:**

Food insecurity was associated with greater odds of non-retention in care and viral non-suppression among PLWHA. Nutritional and mental health pathways are important mediators of these relationships. Results highlight the need for interventions to target these pathways to address food insecurity as an underlying mechanism influencing engagement in HIV care.

## 1. Introduction

In 2014, The Joint United Nations Programme on HIV/AIDS (UNAIDS) initiated its 95-95-95 fast-track strategies to end the HIV epidemic by 2030 (1). The United States (U.S.) National HIV/AIDS Strategy (2022–2025) has introduced similar goals for ending the HIV epidemic by 2030 (2). One such goal is to improve HIV-related outcomes by optimizing engagement in the HIV care continuum (2). The HIV care continuum is a public health framework that describes individual-and population-level stages of HIV care, including diagnosis, linkage to care, receipt of care, retention in care, and achievement and maintenance of viral suppression (3, 4). Successful engagement in the HIV care continuum is essential to ending the HIV epidemic. Poor HIV care engagement is associated with increased HIV viral loads (5) and is a significant driver of the HIV epidemic, as nearly two-thirds of all new infections in the U.S. are transmitted by people living with HIV/AIDS (PLWHA) not retained in care (6, 7). Current national estimates suggest that in 2020, more than 80% of PLWHA were linked to care within 1 month of diagnosis, 74% were engaged in at least one aspect of HIV care, 51% were retained in care, and 65% successfully attained viral suppression. However, such estimates fall short of U.S. National HIV/AIDS Strategy targets to increase care engagement and viral suppression to 95% by 2030 (2). Attaining targets to end the HIV epidemic by 2030 will require a greater understanding of the individual, societal, and structural mechanisms influencing engagement in care among PLWHA. One such mechanism is food insecurity, defined as lacking consistent access to nutrient-rich foods due to a deficiency of resources or the inability to attain food in socially acceptable ways (8, 9).

Worldwide, more than 1 billion people are affected by food insecurity (10). In 2021, approximately 10% of U.S. households experienced food insecurity (11). Compared to the overall U.S. population, food insecurity has disproportionately impacted PLWHA. It is estimated that 25–80% of PLWHA have reported instances of food insecurity (12–14). Food insecurity has also been associated with several clinical outcomes important for HIV care, including lower CD4 cell counts (15, 16), incomplete viral suppression (16–20), and reduced antiretroviral therapy (ART) adherence (21–23). Further, food insecurity has been associated with more frequent acute care utilization (24, 25) and increased mortality (26, 27) among PLWHA. Several studies have examined risk factors for food insecurity among PLWHA (28, 29). Behavioral and mental health factors, such as substance use (29, 30) and depressive symptoms (28), along with socioeconomic factors, such as low income (30–32), unemployment (31), and unstable housing (31, 32), have been identified as risk factors for food insecurity among PLWHA.

Several models have hypothesized the mechanisms through which food insecurity may influence HIV clinical outcomes. For instance, Weiser et al. (10) developed a conceptual framework that explains such effects through nutritional, behavioral, and mental health pathways. A recent study illustrated that these pathways had pronounced effects on viral suppression and CD4 cell counts among women living with HIV (16). Path analyses illustrated that nutritional and behavioral mechanisms accounted for 2% and 31% of the total effect of food insecurity on viral suppression (16). Mental health and behavioral mechanisms accounted for 15% and 17% of the total effect of food insecurity on CD4 cell count (16). While previous work has assessed paths between food insecurity and HIV clinical outcomes, including viral suppression, CD4 cell count, and physical health status (10, 16), few studies have examined the effects of food insecurity on HIV care continuum outcomes (33) and relevant biopsychosocial pathways. Given previous qualitative work has illustrated the impact of behavioral, mental health, and nutritional mechanisms on engagement in HIV care (34, 35), knowledge of the associations between food insecurity and care continuum outcomes and how intervenable behavioral, mental health, and nutritional mechanisms may influence these relationships will allow researchers to develop strategic interventions that target specific pathways based on the intended outcome.

The purpose of this study was to examine the associations between food insecurity and three outcomes in the HIV care continuum. Specifically, we sought to understand the role that food insecurity plays in receipt of care, retention in care, and viral suppression and examine the effects of behavioral, mental health, and nutritional mechanisms through which food insecurity may impact these outcomes. The research questions are (1) Does food insecurity influence HIV care continuum outcomes? and (2) Do nutritional, behavioral, and mental health mechanisms mediate the associations between food insecurity and HIV care continuum outcomes? We hypothesized that food insecurity would be significantly associated with receipt of care, retention in care, and viral suppression. Adapting the conceptual framework describing the bidirectional link between food insecurity and HIV/AIDS (10), we hypothesized that behavioral, mental health, and nutritional mechanisms would mediate the relation between food insecurity and HIV care continuum outcomes.

## 2. Materials and methods

### 2.1. Conceptual framework

**Figure 1:**
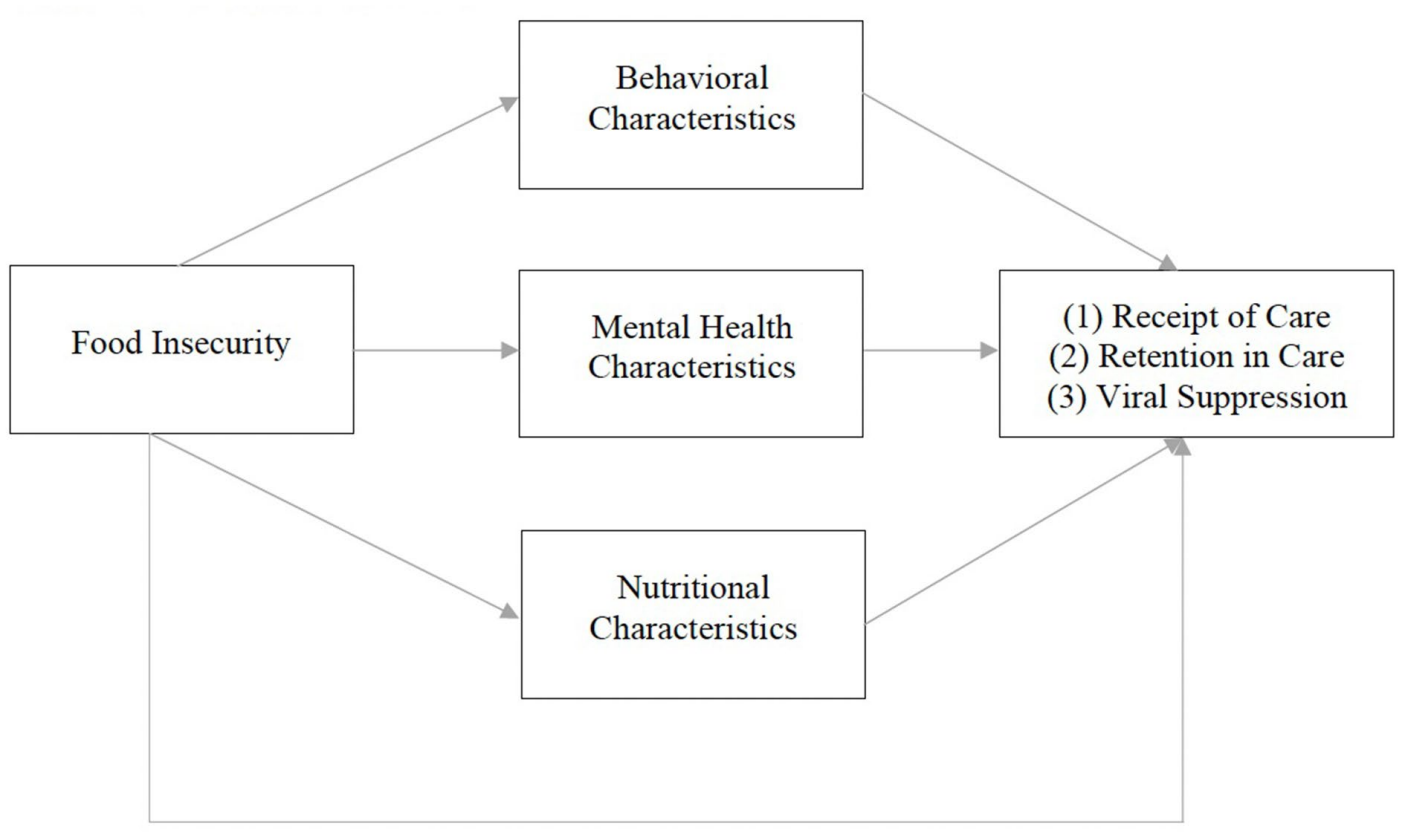
Conceptual framework.

Our conceptual framework is adapted from Weiser et al. (10). The original framework illustrates a bidirectional relationship between food insecurity and HIV morbidity and mortality. It theorizes that food insecurity influences HIV-related outcomes (CD4 cell count and viral suppression) through behavioral (ART non-adherence, missed clinic visits, treatment interruptions), mental health (anxiety, depression, drug/alcohol use), and nutritional (micro/macronutrient deficiencies, ART/food interactions, obesity, lipodystrophy) mechanisms (10). Given previous qualitative work (34), our modified framework posits that food insecurity influences HIV care continuum outcomes (receipt of care, retention in care, viral suppression) via similar behavioral, mental health, and nutritional mechanisms, described below (Figure 1).

### 2.2. Participants and procedures

From May–August 2022, we conducted a web-based, cross-sectional study among a sample of PLWHA in New York State (*N* = 200). Participants were eligible to complete the survey if they were: (1) 18 years of age or older, (2) English speaking, (3) living with HIV, (4) currently residing in New York State, (5) currently prescribed ART for at least 30 days, (6) received food assistance in the past 30 days, (7) had a valid email address, and (8) had access to a smartphone, computer, or tablet to complete the survey. Consistent with our previous work (34), food assistance was defined as receiving Supplemental Nutrition Assistance Programme benefits or visiting a food pantry in the past 30 days.

Participants were recruited using convenience sampling methods. First, study flyers were distributed to local health clinics in Western New York with a history of providing HIV care. Second, study flyers were distributed to the New York State-wide Ending the Epidemic listserv, encouraging members to distribute the study flyer within their networks. Interested persons who responded to the recruitment flyer completed an eligibility screener and a brief demographic survey. The Principal Investigator (PI) reviewed each respondent’s screener to determine eligibility. If eligible, the PI sent the participant a unique link via email to complete an online, self-administered survey at a time and location that was most convenient for them. Upon survey completion, participants received a $15 Amazon electronic gift card. To avoid duplicate study participants, any respondents with an identical IP address and/or email address were prohibited from completing the survey a second time (*n* = 1). To estimate the necessary sample size, we followed criteria set forth by Fritz and MacKinnon (36) to defect small to moderate effects for indirect paths in mediation models employing bias-corrected bootstrapping (36). All procedures were in accordance with the ethical standards of the 1964 Helsinki declarations and its later amendments. The study protocol was approved by the University at Buffalo Institutional Review Board with a waiver for written informed consent (STUDY00006193).

### 2.3. Measures

We administered the survey using Research Electronic Data Capture (REDCap) and collected anonymous, self-reported information regarding participant’s demographic characteristics, food security status, HIV care continuum outcomes, and various behavioral, mental health, and nutritional characteristics.

#### 2.3.1. Primary exposure variable

Past-month (30 days) food insecurity was measured using questions from the U.S. Department of Agriculture’s Household Food Security Survey Module, the U.S. Adult Food Security Scale, and the U.S. Children’s Food Security Scale (37). As outlined in the U.S. Household Food Security Survey Module, all participants received three household-level questions pertaining to food security: (1) *“We worried whether our food would run out before we got money to buy more. Was that often true, sometimes true, or never true for you and your household in the last 30 days?*”, (2) *“The food that we bought just did not last, and we did not have money to get more. Was that often, sometimes, or never true for you or your household in the last 30 days?”*, and (3) *“We could not afford to eat balanced meals. Was that often, sometimes, or never true for you or your household in the last 30 days?”* After the completion of these questions, participants who did not report child dependents under the age of 18 completed the U.S. Adult Food Security Scale. Participants who indicated having child dependents under the age of 18 were administered the U.S. Children’s Food Security Scale (37). Affirmative answers were summed for each participant to create a total food security score as a continuous variable, where higher scores indicated increasing severity of food insecurity. A score of 0 indicated high food security, a score of 1–2 indicated marginal food security, a score of 3–5 indicated low food security, and a score above 6 indicated very low food security (37).

#### 2.3.2. Primary outcome variables

The primary outcome variables were three stages in the HIV care continuum, as defined by the Centers for Disease Control and Prevention (38). These included: (1) self-report receipt of care (persons with diagnosed HIV who had at least one CD4 or viral load test), (2) self-report retention in care (persons with diagnosed HIV who had two or more CD4 or viral load tests, performed at least 3 months apart), and (3) self-report viral suppression (viral load test result of <200 copies/mL at the most recent viral load test during measurement year). Each outcome measure was measured as a dichotomous variable (yes/no).

#### 2.3.3. Sociodemographic characteristics

Data were collected on participant’s age, race, ethnicity, sex assigned at birth, current gender identity, sexual orientation, educational attainment, employment status, annual household income, housing status, marital status, and year of HIV diagnosis.

#### 2.3.4. Behavioral characteristics

We collected data on various HIV-related behavioral characteristics, including missed HIV clinic visits (defined as missed HIV clinical visits that were not rescheduled within the past year) and past 30-day ART adherence (visual analog scale). Optimal ART adherence was then operationalized as ≥95% (yes/no).

#### 2.3.5. Mental health characteristics

We collected data on various mental health characteristics. *Depression*. Depression was measured using the Center for Epidemiologic Study Depression Scale (CES-D) (39). The CES-D consists of 20 items that assess past week depressive symptoms, such as feelings of loneliness, sleeplessness, and poor appetite on a four-point scale, ranging from “rarely or none of the time” to “most or all of the time” (Cronbach’s *α* = 0.96 [all Cronbach’s *α* denote measurement in the current study]). *Anxiety*. Anxiety was measured using the Generalized Anxiety Disorder-7 (GAD-7) (40). The GAD-7 consists of seven items that assess past two-week anxiety symptoms, such as feeling nervous or on edge, worrying too much, and trouble relaxing (Cronbach’s *α* = 0.94). *Perceived stress.* Perceived stress was measured using the 10-item Perceived Stress Scale (PSS) (41). Items assessed the degree to which certain scenarios are appraised as stressful on a five-point scale ranging from “never” to “very often” (Cronbach’s *α* = 0.78). *General mental health status.* General mental health status was assessed using the MOS-HIV (42). The mental health dimension of the MOS-HIV consists of 5 items that assess overall mental health status within the past 30 days on a six-point scale, ranging from “all of the time” to “none of the time” (Cronbach’s *α* = 0.82). Lastly, illicit substance use was measured as self-reported cocaine, crack, methamphetamine, sedative/sleeping pills, hallucinogens, street opioids, prescription opioids, and other illicit drug use within the past 3 months (yes/no).

#### 2.3.6. Nutritional characteristics

Participants were asked to self-report their height (inches) and weight (pounds). Body Mass Index (BMI) was calculated from anthropometric measures using the following equation: $\frac{Weight\;\left(\mathrm{lb}\right)}{{\left[height\;\left(in\right)\right]}^{2}}\times 703$ (43).

### 2.4. Statistical analyses

All statistical analyses were conducted using Stata 17.0 (StataCorp LP, College Station, TX). We conducted descriptive statistics (medians with interquartile ranges [IQR] for continuous variables and frequency distributions with percentages for categorical variables) on all variables of interest. We then conducted Chi square (for categorical variables) and Mann–Whitney U tests (for continuous variables) to examine if variables differed significantly for each outcome measure. To test research question 1, we conducted two individual multivariable logistic regression models to estimate adjusted odds ratios (aOR) and 95% Confidence Intervals (CI) for the associations between food security status and (1) retention in care and (2) viral suppression, adjusting for age, race, ethnicity, education, income, and marital status. Given the large percentage of participants who reported receipt of care (*n* = 187, 95.9%), we did not conduct any inferential statistics for this outcome variable.

To examine research question 2, we conducted individual causal mediation analyses (44) to determine whether behavioral (optimal ART adherence, missed HIV clinic appointments), mental health (depression, anxiety, perceived stress, mental health status, illicit substance use) and nutritional mechanisms (BMI) mediated the relationships between food security status and HIV care continuum outcomes. Causal mediation analyses have been shown to be more robust against modeling misspecifications, such as non-linearity and interactions (44, 45). Developed by Valeri and VanderWeele (45), we used the Stata command “paramed” to estimate the odds ratio for natural direct (OR_NDE_) and natural indirect (OR_NIE_) effects and 95% CIs by fitting logistic regression models to assess the relationships between food security status and each outcome variable, adjusting for age, race, ethnicity, education, income, and marital status. We selected the strongest and most significant (*p* < 0.05) mediator along each path for our final models. For the behavioral path, missed HIV clinic visits was the strongest mediator for non-retention in care and optimal ART adherence was the strongest mediator for viral non-suppression. BMI and depression were the strongest mediators for the nutritional and mental health pathways, respectively, for both non-retention in care and viral non-suppression.

## 3. Results

### 3.1. Participant characteristics

Of the 200 PLWHA in our study, the median age was 30 years (IQR: 27–37 years). The majority of participants self-identified as Black (54.0%), non-Hispanic (89.0%), male (55.5%) and straight/heterosexual (63.0%). More than half of participants reported being unemployed (60.5%) and having an annual household income under $10,000 (57.5%). The median food security score was 9 (IQR: 6–10), corresponding to very low food security. Additional participant characteristics are presented in Table 1.

**Table 1:** Demographic and nutritional, behavioral and mental health correlates of HIV care continuum engagement among a sample of PLH in New York State (*N*=200).

		Receipt of Care			Retention in Care			Viral Suppression		
Characteristics	Total (*N*=200) *n* (%) or Median (IQR)	*Receipt of care* (*N* = 187) *n* (%) or Median (IQR)	*No receipt of care* (*N* = 8) *n* (%) or Median (IQR)	*p*-value	*Retention in care* (*N* = 144) *n* (%) or Median (IQR)	*Non-retention in care* (*N* = 49) *n* (%) or Median (IQR)	*p*-value	*Viral suppression* (*N* = 102) *n* (%) or Median (IQR)	*Viral non-suppression* (*N* = 69) *n* (%) or Median (IQR)	*p*-value
Food security status (0–10), Median (IQR)	9 (6–10)	9 (6–10)	10 (9.5–10)	0.100	8 (5.5–10)	10 (9–10)	<0.001	7 (4.5–10)	10 (9–10)	<0.001
Age (years)	30 (27–37)	30 (27–34)	28.5 (27–30)	0.398	30 (27–34)	29 (27–33)	0.303	30 (26–35)	30 (27–33)	0.829
Race				0.572			0.170			0.411
White	69 (34.5)	66 (35.3)	2 (25.0)		54 (37.5)	14 (28.6)		39 (38.2)	21 (30.4)	
Black	108 (54.0)	100 (53.5)	6 (75.0)		72 (50.0)	31 (63.3)		51 (50.0)	42 (60.9)	
Other	23 (11.5)	21 (11.2)	0 (0.0)		18 (12.5)	3 (6.1)		12 (11.8)	6 (12.2)	
Ethnicity^a^				0.366			0.163			0.052
Non–Hispanic/Latino	178 (89.0)	166 (88.8)	8 (100)		125 (86.8)	47 (95.9)		89 (87.3)	67 (97.1)	
Hispanic/Latino	18 (9.0)	17 (9.1)	0 (0.0)		15 (10.4)	2 (4.1)		11 (10.8)	2 (2.9)	
Sex assigned at birth^a^				0.607			0.751			0.264
Male	116 (58.0)	110 (58.8)	4 (50.0)		86 (59.7)	28 (57.1)		62 (60.8)	36 (52.2)	
Female	83 (41.5)	76 (40.6)	4 (50.0)		58 (40.3)	21 (42.9)		40 (39.2)	33 (47.8)	
Gender^a^				0.757			0.789			0.351
Man	111 (55.5)	105 (56.1)	4 (50.0)		81 (56.3)	28 (57.1)		58 (58.9)	35 (52.2)	
Woman	81 (40.5)	74 (39.6)	4 (50.0)		57 (39.6)	20 (40.8)		39 (38.2)	32 (46.4)	
Transgender or genderqueer	7 (3.5)	7 (3.7)	0 (0.0)		6 (4.1)	1 (2.0)		5 (4.9)	1 (1.5)	
Sexual identity				0.159			0.385			0.472
Straight	126 (63.0)	118 (63.10)	7 (87.5)		90 (62.5)	34 (69.4)		69 (67.7)	43 (62.3)	
Lesbian/Gay/Bisexual/Queer	74 (37.0)	69 (36.9)	1 (12.5)		54 (37.50)	15 (30.61)		33 (32.4)	26 (37.7)	
Education^a^				0.358			0.032			0.001
High school degree or lower	40 (20.0)	37 (19.8)	0 (0.0)		32 (22.2)	4 (8.16)		22 (21.6)	2 (2.9)	
Some college and higher	155 (77.5)	145 (77.5)	8 (100.0)		109 (75.7)	43 (87.8)		79 (77.5)	65 (94.2)	
Employment status^a^^,^ ^b^				0.125			<0.001			<0.001
Employed	77 (38.5)	73 (39.0)	1 (12.5)		67 (46.5)	6 (12.2)		49 (48.0)	9 (13.0)	
Unemployed	121 (60.5)	112 (59.9)	7 (87.5)		76 (52.7)	42 (85.7)		53 (52.0)	60 (97.0)	
Annual household income^a^				0.163			<0.001			<0.001
<$10,000	115 (57.5)	107 (57.2)	7 (87.5)		72 (50.0)	41 (83.7)		54 (52.9)	59 (85.5)	
≥$10,000	66 (33.0)	62 (33.2)	1 (12.5)		59 (41.0)	4 (81.6)		42 (41.2)	7 (10.1)	
Housing status^a^				0.232			<0.001			<0.001
Stable	63 (31.5)	60 (32.1)	1 (12.5)		55 (38.2)	5 (10.2)		37 (36.3)	7 (10.1)	
Unstable	134 (67.0)	124 (66.3)	7 (87.5)		87 (60.4)	43 (87.8)		65 (63.7)	59 (85.5)	
Marital status^a^				0.598			0.932			0.461
Never married	80 (40.0)	75 (40.1)	2 (25.0)		57 (39.6)	19 (38.8)		45 (44.1)	24 (34.8)	
Married	63 (31.5)	59 (31.6)	4 (50.0)		46 (31.9)	17 (34.7)		32 (31.4)	26 (37.7)	
Divorced/Separated/Widowed	55 (27.5)	52 (27.8)	2 (25.0)		41 (28.5)	13 (26.5)		25 (24.51)	19 (27.54)	
Years living with HIV	4 (3–6)	4 (3–6)	3 (2–3.5)	0.178	4 (2–7)	4 (3–5)	0.234	5 (3–8)	3 (3–6)	0.353
Missed HIV clinic visits (past year)^a^				0.027			0.105			0.028
No	99 (49.5)	96 (51.3)	1 (12.5)		76 (52.8)	20 (40.8)		56 (54.9)	25 (36.2)	
Yes	97 (48.5)	87 (46.5)	7 (87.5)		64 (44.4)	29 (59.2)		45 (44.1)	41 (59.4)	
Optimal ART adherence ( ≥ 95%)				0.066			0.012			0.058
No	89 (44.5)	85 (45.5)	1 (12.5)		71 (49.3)	14 (28.6)		48 (47.1)	22 (31.88)	
Yes	111 (55.5)	102 (54.5)	7 (87.5)		73 (50.7)	35 (71.4)		54 (52.9)	47 (68.1)	
Depression^a^ (0–27), Median (IQR)	32 (20–52)	32 (19–52)	51.5 (49.5–53)	0.057	26.5 (19–49)	52 (40–54)	<0.001	28 (18–44.5)	52 (48–54)	<0.001
Anxiety (0–21), Median (IQR)	13 (7–18)	13 (7–18)	19.5 (16.5–20)	0.018	11 (6–16)	17 (15–19)	<0.001	11 (7–16)	17 (15–19)	<0.001
Perceived stress^a^ (0–40), Median (IQR)	23 (17–27)	23 (17–27)	28 (25.5–29)	0.012	21 (16–26)	26 (24–28)	<0.001	21 (16–26)	26 (23–28)	<0.001
Mental health status (5–30), Median (IQR)	16 (14–20)	16 (13–20)	14.5 (14–15.5)	0.159	17 (14–21)	14 (12–16)	<0.001	18 (14–20)	14 (11–16)	<0.001
Illicit substance use				0.419			0.126			0.254
No	182 (91.9)	171 (91.4)	8 (100.0)		134 (93.1)	42 (87.8)		92 (90.2)	66 (95.7)	
Yes	16 (8.1)	14 (7.5)	0 (0.0)		8 (5.6)	6 (12.2)		9 (8.8)	3 (4.35)	
BMI, Median (IQR)	26.4 (23.2–34.8)	26.01 (23.2–34.6)	35.8 (33.1–37.1)	0.004	25.7 (22.9–33.0)	34.9 (25.8–37.2)	<0.001	25.7 (22.7–29.9)	34.5 (24.6–37.0)	<0.001

a Totals do not sum to 100% due to some participants refusing to answer.

b Employment was operationalized as part-time or full-time work.

### 3.2. Non-retention in care

The majority of participants reported retention in HIV care (72.0%). Unadjusted models illustrated a significant association between increasing severity of food insecurity and increased odds of non-retention in care. In adjusted models, increasing severity of food insecurity was associated with 1.35 (95% CI: 1.07, 1.70) higher odds of non-retention in care compared to participants with lower severity of food insecurity (Table 2). For the nutritional path, mediation analyses illustrated an indirect relationship (natural indirect effect) of food insecurity on non-retention in care mediated (31.2% of the total effect) through BMI (OR_NIE_: 1.08, 95% CI: 1.00, 1.18). For both the behavioral and mental health paths, there was no evidence of an indirect relationship of food insecurity on non-retention in care mediated by missed HIV clinic visits (OR_NIE_: 0.99, 95% CI: 0.97, 1.00) or depression (OR_NIE_: 1.14, 95% CI: 0.94, 1.35), respectively (Table 3).

**Table 2:** Crude and adjusted odds ratios (aOR) from multivariable logistic regression models assessing associations between food security status and HIV care continuum outcomes.

Exposure	Outcome	Crude OR	95% CI	aOR^a^	95% CI
Food security status					
	Non-retention in care	1.29**	1.10, 1.52	1.35*	1.07, 1.70
	Viral non-suppression	1.26**	1.11, 1.43	1.29*	1.08, 1.54

*p<0.01; ^**^p<0.001.

a Models adjusted for age, race, ethnicity, education, income, and marital status.

**Table 3:** Estimates of direct, indirect, and total effects of nutritional, behavioral, and mental health paths from food insecurity to non-retention in care and viral non-suppression.

	Non-retention in care			Viral non-suppression		
	OR	95% CI^a^	% Mediated^b^	OR	95% CI^a^	% Mediated^b^
Behavioral path^c^						
Natural direct effect	1.38*	1.09, 1.77		1.31*	1.09, 1.62	
Natural indirect effect	0.99	0.97, 1.00		1.00	0.98, 1.02	
Total effect	1.36*	1.10, 1.74		1.31*	1.07, 1.59	
Mental health path: depression						
Natural direct effect	1.13	0.78, 1.62		0.93	0.67, 1.28	
Natural indirect effect	1.14	0.94, 1.35		1.27*	1.07, 1.47	100%
Total effect	1.28*	1.02, 1.58		1.19*	0.95, 1.37	
Nutritional path: BMI						
Natural direct effect	1.19	0.92, 1.58		1.18	0.98, 1.46	
Natural indirect effect	1.08*	1.00, 1.18	31.18%	1.07*	1.00, 1.16	27.41%
Total effect	1.28*	1.02, 1.61		1.28*	1.03, 1.49	

Individual path models controlled for age, race, ethnicity, income, education, and marital status. *p<0.05.

a Bootstrapped confidence interval with 1000 replications.

b Calculated as log(OR_natural indirect effect_)/log(OR_total effect_).

c Missed HIV clinic visits was the primary mediator investigated for non-retention in care. Optimal ART adherence was the primary mediator investigated for viral non-suppression.

### 3.3. Viral non-suppression

More than half of participants reported viral suppression (51%). Increasing severity of food insecurity was associated with increased odds of viral non-suppression in unadjusted models. In adjusted models, increasing severity of food insecurity was associated with 1.29 (95% CI: 1.08, 1.54) higher odds of viral non-suppression compared to participants with less severe food insecurity (Table 2). There was an indirect relationship for nutritional pathways (OR_NIE_: 1.07, 95% CI: 1.00, 1.16) mediated (27.4% of the total effect) through BMI and an indirect relationship for mental health pathways (OR_NIE_: 1.27, 95% CI: 1.07, 1.47) mediated (100.0% of the total effect) through depression. For the behavioral path, there was no evidence of an indirect relationship of food insecurity on viral non-suppression mediated through optimal ART adherence (OR_NIE_: 1.00, 95% CI: 0.98, 1.02) (Table 3).

## 4. Discussion

In this cross-sectional study of 200 PLWHA, food insecurity was associated with greater odds of non-retention in care and viral non-suppression. Our findings illustrated that nutritional and mental health pathways indirectly mediated the relationship between food insecurity and worse HIV care continuum outcomes. To our knowledge, this is one of the first studies to estimate the effects of food insecurity on various care continuum outcomes and to examine potential mediators within those relationships. Our findings underscore the need to address food insecurity among PLWHA and highlight the need to integrate food assistance interventions into comprehensive HIV care.

Our findings are consistent with previous work illustrating the relationship between food insecurity and worse HIV outcomes (14, 16, 19, 20, 33, 46). However, the prevalence of food insecurity among PLWHA in our sample was greater than other studies (16, 19, 47), with approximately 78% reporting very low food security and nearly 95% of our sample reporting any food insecurity. For instance, the prevalence of very low food security in the Women’s Interagency HIV Study was approximately 14% (16, 19). Similarly, the prevalence of any food insecurity among a sample from the Veterans Aging Cohort Study was 24% (47). The higher prevalence of food insecurity among our sample is likely a result of the COVID-19 pandemic. Recent literature has illustrated that food insecurity among PLWHA has been exacerbated by the pandemic (48, 49). One qualitative study conducted among PLWHA illustrated how food insecurity as a result of the COVID-19 pandemic influenced engagement in HIV care via mental health pathways (34), a result consistent with our study. Bleasdale et al. (34) found that pandemic-related food insecurity led to feelings of anxiety and depression, which impacted ART adherence and care engagement. Future studies should consider the enduring effects of the COVID-19 pandemic on food insecurity and HIV care among PLWHA.

Neither missed HIV clinic visits or optimal ART adherence mediated the relationships between food insecurity and non-retention in care and viral non-suppression, respectively. While consistent with previous work (47), this finding is in contrast with a current study identifying optimal ART adherence (≥95%) adherence as a strong, significant mediator in the relationship between food insecurity and viral non-suppression (16). It is possible that the cross-sectional and self-report nature of our data explains this divergence. Self-report ART adherence is subjected to recall bias and may not reflect the full scope of adherence behaviors, such as medication and treatment interruptions (50). Further, it is likely that food insecurity has independent effects on the pharmacokinetics of several antiretroviral medications that require food for optimal absorption (22, 51). While we did not collect information regarding specific ART regimens, it is possible that food insecurity contributes to poor drug absorption and viral non-suppression, despite high self-report ART adherence (22). Future studies may consider the use of objective measures of adherence beyond self-reported data.

Consistent with previous work (16), depression was the most salient mental health mediator for both non-retention in care and viral non-suppression. Quantitative studies have demonstrated food insecurity to be significantly associated with psychological distress among PLWHA (52–54). A recent cross-sectional study of 1,305 women living with HIV indicated a significant, dose–response relationship between food insecurity and depressive symptoms (54). In previous qualitative work, feelings of worry, anxiety, and depression as a result of food insecurity negatively impacted care engagement for PLWHA (34, 35). Recent quantitative work has illustrated marginally lower prevalence of viral suppression among PLWHA with mental health concerns compared to PLWHA without mental health concerns (55). Our results add to the burgeoning literature suggesting the need for interventions that simultaneously address food insecurity and mental health concerns (16, 56). Coupling food insecurity interventions with mental health counseling or integrating food insecurity interventions into comprehensive care for PLWHA may be most effective at increasing care engagement for PLWHA.

Our results illustrated a significant indirect effect of nutritional pathways on the relationships between food insecurity and non-retention in care and viral non-suppression, a finding consistent with previous qualitative research (16, 34, 35). Yet, our findings illustrated a stronger indirect effect of the nutritional pathways compared to previous quantitative work (16). These differing results highlight the need to use more rigorous methodologies to ascertain nutritional data among PLWHA. Similar to previous work (16) and guided by our adapted conceptual framework (10), anthropometric measurements (BMI) represented the nutritional paths for each outcome. Yet, research has illustrated BMI to be a poor indicator of overall health (57). Other studies examining nutritional pathways as potential mediators in the relationships between food insecurity and other HIV outcomes (e.g., CD4 cell count, physical health status) have used food intake from food frequency questionnaires (16). While food frequency questionnaires are more time efficient and allow for longer term intake compared to other methods (58), self-reported food frequency questionnaires are subject to recall and social desirability biases (58). One potential solution could be the use of more rigorous intake methods, such as remote food photography. Remote food photography is a valid, gold-standard measure that utilizes photographs to capture planned food consumption (pre-meal) and plate waste (post-meal) to provide estimates of energy, micro- and macro-nutrient intake (59). The integration of such methodologies may provide a more nuanced understanding of the nutritional mechanisms that mediate the relationships between food insecurity and HIV-related outcomes.

Our results suggest the need to integrate food insecurity interventions into HIV care. While there is a growing body of literature examining the feasibility and effectiveness of food assistance interventions on HIV-related outcomes globally (60–62), few studies have extended this work to high-income countries, such as the U.S. (63–65). Palar et al. (64) found that very low food security significantly decreased following the implementation of a food support intervention for PLWHA in San Francisco, California. Results also indicated that participants were less likely to sacrifice healthcare for food and more likely to be ART adherent (64). Given our results, future studies should continue to evaluate the effectiveness of food assistance interventions on HIV outcomes and seek to understand key stakeholders’ (i.e., healthcare and social service providers, clinic directors) perspectives on integrating food insecurity and food access interventions into comprehensive HIV care.

This study had several strengths including a diverse sample, representation of women, and the use of robust psychosocial measures. Further, to our knowledge, this is one of the first studies to assess the influence of food insecurity on HIV care continuum outcomes beyond viral suppression. However, our study should be interpreted within the context of its limitations. First, we were unable to perform meaningful statistical inferences on our first outcome measure (receipt of care) due to the large affirmative response rate. Second, our study relied on a convenience sample from one state in the U.S.; therefore, our findings may not be generalizable to PLWHA who reside in other geographical areas. Third, participants who reported child dependents under the age of 18 were only administered the U.S. Children’s Food Security Scale. As such, responses from the U.S. Children’s Food Security Scale were used to estimate food security scores for these participants, which may limit the reliability of study results. Future research should confirm study results using validated measures. Fourth, our data was cross-sectional which precludes inferences of causality. Yet, our findings from the causal mediation analyses are similar to results from longitudinal studies (16). Fifth, participants’ responses were subjected to recall and social desirability bias as responses were self-reported and not confirmed with pharmacy or medical records. Lastly, our relatively small sample size may present additional limitations. Rigorously designed intervention studies with larger samples are needed to confirm study results. Yet, despite these limitations, our study offers valuable insights into food insecurity as an underlying mechanism influencing care engagement among PLWHA and potential mechanisms for intervention.

In conclusion, our study illustrated that food insecurity is associated with greater odds of non-retention in care and viral non-suppression and that these relationships are influenced via nutritional and mental health pathways. Future studies should continue to examine these relationships with objective biological measures. Such investigations will help to inform the development of socio-structural- and policy-level interventions to address food insecurity among PLWHA in an effort to attain U.S. and UNAIDS goals to end the HIV epidemic by 2030.

## Data availability statement

The original contributions presented in the study are included in the article/supplementary material, further inquiries can be directed to the corresponding author.

## Ethics statement

The studies involving human participants were reviewed and approved by University at Buffalo Institutional Review Board. Written informed consent for participation was not required for this study in accordance with the national legislation and the institutional requirements.

## Author contributions

JB and SP: conceptualization and methodology. JB and YL: formal analysis and investigation. JB: writing—original draft preparation, funding acquisition, and resources. JB, YL, GM, LL, and SP: writing—review and editing. SP: supervision. All authors have read and agreed to the published version of the manuscript.

## Funding

This study was supported The Mark Diamond Research Fund of the Graduate Student Association at the University at Buffalo, the State University of New York.

## Conflict of interest

The authors declare that the research was conducted in the absence of any commercial or financial relationships that could be construed as a potential conflict of interest.

## Publisher’s note

All claims expressed in this article are solely those of the authors and do not necessarily represent those of their affiliated organizations, or those of the publisher, the editors and the reviewers. Any product that may be evaluated in this article, or claim that may be made by its manufacturer, is not guaranteed or endorsed by the publisher.
